# CT-based explainable machine learning for predicting benign and malignant thyroid nodules: a multi-center study

**DOI:** 10.3389/fonc.2025.1675943

**Published:** 2025-12-09

**Authors:** Haijun He, Mingquan Luo, Kai Hu, Tengfei Ke, Juntao Yang, Xinyue Zi, Tingting Jiang, Liangping Yong, Tong Chen, Jun Chen, Zhengliang He, Qiangrong Gao, Zhoubin Liang, Yang Jing, Bin Yang

**Affiliations:** 1 Department of Radiology, Nanbu County People’s Hospital, Nanchong, Sichuan, China; 2 Yunnan Cancer Hospital, The Third Affiliated Hospital of Kunming Medical University, Peking University Cancer Hospital, Kunming, China; 3 Department of Radiology, Dali Bai Autonomous Prefecture People’s Hospital, Dali, Yunnan, China; 4 Department of Radiology, The First Affiliated Hospital of Dali University, Dali, China; 5 Department of Blood Transfusion, Nanbu County People's Hospital, Nanchong, Sichuan, China; 6 Huiying Medical Technology Co., Ltd, Beijing, China; 7 Medical Image Center, Kunming Medical University Affiliated Calmette Hospital, Kunming, China

**Keywords:** thyroid nodules, benignity or malignancy, CT, machine learning, SHapley additive explanations

## Abstract

**Objective:**

This study intends to construct a CT-based explainable machine learning model for preoperative prediction of thyroid nodule benignity or malignancy, aiming to provide a more accurate tool for clinical decision-making and management.

**Materials and methods:**

A retrospective study included 370 patients with thyroid nodules confirmed by pathology from three centers, divided into a training set (*n =* 229) and an internal validation set (*n =* 100) in a 7:3 ratio, with patients from the third center serving as an external validation set (*n =* 41). Radiomics features were extracted from preoperative CT images, and the optimal features were selected to construct a radiomics score (Rad_Score). Clinical risk factors were identified using univariate and multivariate logistic regression. LR and SVM algorithms were used to establish three models: a clinical model, an imaging model, and a combined model (integrating clinical factors and Rad_Score). The combined model was visualized using SHAP (SHapley Additive exPlanations) analysis. Model performance was evaluated using receiver operating characteristic (ROC) curves, calibration curves, and decision curve analysis (DCA).

**Results:**

A total of 17 features were ultimately selected for Rad_Score calculation. The combined model demonstrated the best performance, with the LR combined model achieving AUC values of 0.962, 0.913, and 0.914 in the training set, internal validation set, and external validation set, respectively, all higher than the LR clinical model and LR radiomics model; and the LR combined model outperforms the SVM combined model (0.953, 0.885, and 0.842). The SHAP analysis revealed the relative importance of the key feature (Rad_score) in model prediction, enhancing model transparency.

**Conclusion:**

The combined model performs better under the LR algorithm. Combined with SHAP explainable analysis, it provides a non-invasive, efficient, and transparent tool for preoperative differentiation of benign and malignant thyroid nodules, potentially optimizing individualized clinical management.

## 1 Introduction

Thyroid nodules are a common clinical condition (1), with an incidence rate of approximately 5%–7% in adults. Approximately 10% of nodules are thyroid cancer (2, 3), which is particularly prevalent in women. In China, it has become one of the five most common cancers in women, and its incidence rate continues to rise (4). In recent years, the widespread use of CT scans has significantly increased the incidental detection rate of thyroid nodules (5). However, there is currently no unified guideline for the management of incidental nodules. The American Thyroid Association (ATA) recommends further evaluation for nodules ≥1 cm, but relying solely on morphological characteristics to distinguish between benign and malignant nodules has limitations; for nodules <1 cm, evaluation is only necessary when accompanied by clinical symptoms or suspicious enlarged lymph nodes.

Fine-needle aspiration biopsy is currently considered the gold standard for diagnosing benign or malignant thyroid nodules (6), but it is an invasive procedure, and a single aspiration can only obtain a small portion of tumor tissue. Due to tumor heterogeneity, relying solely on small tissue samples cannot fully reflect all tumor information (7). Therefore, some nodules require multiple aspirations, which may lead to the spread of cancer cells (8). The primary techniques currently used to detect thyroid nodules include ultrasound, computed tomography (CT), and magnetic resonance imaging (MRI). Among these, ultrasound is the most widely used method, but its diagnostic results are highly dependent on the operator’s experience and exhibit significant subjectivity (9). In contrast, CT and MRI are unreliable in distinguishing between benign and malignant nodules, with misdiagnosis rates as high as 40%–70% (10). The widespread use of cervical and thoracic CT has established it as a primary pathway for detecting incidental thyroid nodules. Confronted with this large and growing population, there is a pressing clinical need for a method that can perform initial risk stratification directly based on the first-look CT images, thereby guiding subsequent decisions regarding the necessity of dedicated ultrasonography or biopsy. This approach is crucial for efficient patient triage and optimal allocation of healthcare resources. Moreover, CT provides indispensable prognostic information by precisely delineating the relationship between the nodule and adjacent structures (e.g., vessels, trachea) and by comprehensively evaluating cervical lymph nodes (11). Its ability to assess deep-seated lymph nodes that are often beyond the reach of ultrasound is particularly critical, as metastatic involvement is a powerful indicator of malignancy. Surgery is the primary treatment for thyroid cancer, but it has been found that approximately 70%–80% of patients with thyroid nodules undergo unnecessary total or near-total thyroidectomy, resulting in these patients needing to take thyroid hormones for life (12). Therefore, constructing a non-invasive diagnostic model that uses high-precision quantitative analysis of tumor heterogeneity features can overcome the limitations of traditional methods (operator dependency, sampling errors, and subjective morphological assessment), providing objective and quantifiable decision-making criteria for distinguishing between benign and malignant thyroid nodules, which holds significant value for optimizing clinical management. Therefore, the proposed CT-based model is designed not to replace ultrasonography but to form a complementary relationship with it, leveraging the strengths of both modalities to achieve a more efficient and comprehensive diagnostic workflow.

Radiomics is an emerging technology that extracts a large number of quantitative features from medical images and converts these features into data for analysis (13). Its objective is to use computer algorithms and machine learning models to extract more information from conventional medical images, identify key imaging biomarkers that are not detectable by the naked eye, improve diagnostic accuracy, assess treatment responses and prognosis-related issues, and thereby assist clinical decision-making (14–16).

Currently, radiomics studies based on CT for thyroid nodules are not common, and existing related radiomics studies are mostly single-center, small-sample designs, with no analysis of model interpretability. Therefore, this study included CT images from a multi-center, large-sample cohort of thyroid nodule patients, integrating CT radiomics features with independent clinical risk factors to construct and validate a radiomics nomogram model combining both. Additionally, the model underwent SHAP interpretability analysis to assess the contribution and influence of radiomics features and clinical indicators on the model, visually illustrating the model’s decision-making process.

## 2 Materials and methods

### 2.1 Patient data

**Figure 1:**
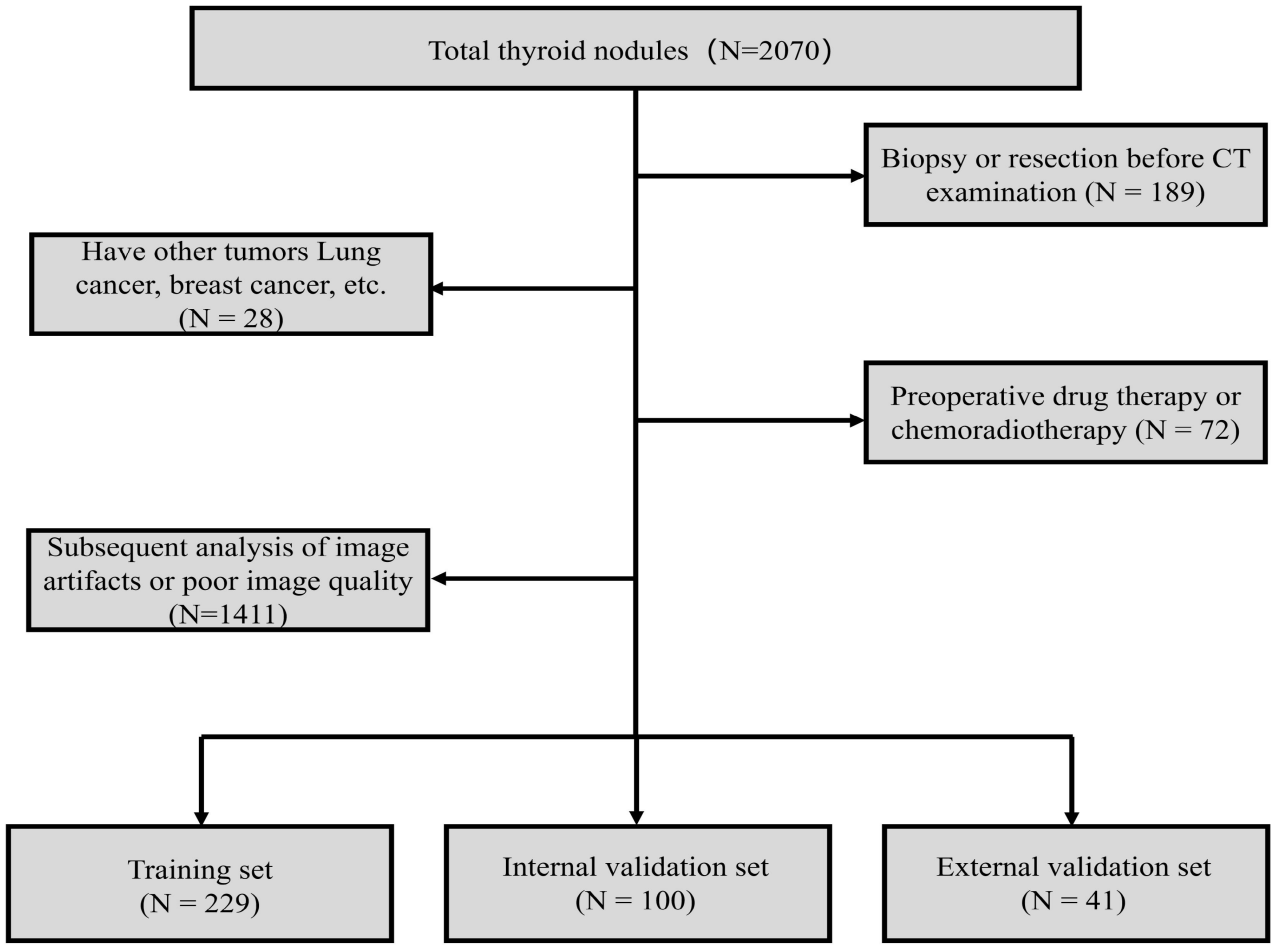
Flowchart of patient inclusion and exclusion criteria.

This retrospective study was approved by the hospital ethics committee and institutional review board, and informed consent was waived. Patients with thyroid nodules who were treated at Dali University First Affiliated Hospital (center 1), Yunnan Provincial Cancer Hospital (center 2), and Dali Prefecture People’s Hospital (center 3) from January 2015 to November 2021 were consecutively enrolled according to the inclusion and exclusion criteria. The inclusion criteria were as follows: (1) pathological confirmation of the surgical specimen, (2) maximum diameter of the thyroid lesion ≥1.0 cm, and (3) complete clinical and CT data. The exclusion criteria were as follows: (1) patients who had undergone biopsy or resection prior to CT examination, (2) patients with other tumor diseases, (3) patients with a history of preoperative drug therapy or neck radiotherapy/chemotherapy, and (4) patients with artefacts or poor image quality affecting subsequent analysis. **Figure 1** shows the patient inclusion and exclusion process. Clinical data for each patient were obtained by reviewing medical records, including gender, age, family history, lesion characteristics (location, number, size, borders, aspect ratio, density, calcification, lobulation/cystic changes), patient blood routine indicators (platelet count, neutrophil count, lymphocyte count, monocyte count), and thyroid function indicators (FT3, FT4, TT3, TT4, TSH, TGAB%). After screening using the exclusion criteria, 370 patients were ultimately included, of whom 329 (218 benign and 111 malignant) were assigned to the training set (229 cases) and internal validation set (100 cases), and 41 were assigned to the external validation set (19 benign and 22 malignant).

### 2.2 CT scanning technology

Dali University First Affiliated Hospital uses a Philips 16-slice CT scanner with the following scan parameters: tube voltage—120 kV, tube current—250 mAs, slice thickness—3 mm, reconstruction slice thickness—1.5 mm, and slice spacing—1.5 mm. Yunnan Provincial Cancer Hospital uses a Philips 64-slice CT scanner with the following scan parameters: tube voltage—120 kV, tube current—300 mAs, and slice thickness—3 mm. Dali Prefecture People’s Hospital uses a Siemens 16-slice CT scanner with the following scanning parameters: tube voltage—120 kV, tube current—250 mAs, and slice thickness—2 mm. Before scanning, the patients must inhale and hold their breath, with both arms placed alongside the body. During scanning, the patients must maintain a supine position with the neck extended backward and must not swallow.

### 2.3 Image segmentation

**Figure 2:**
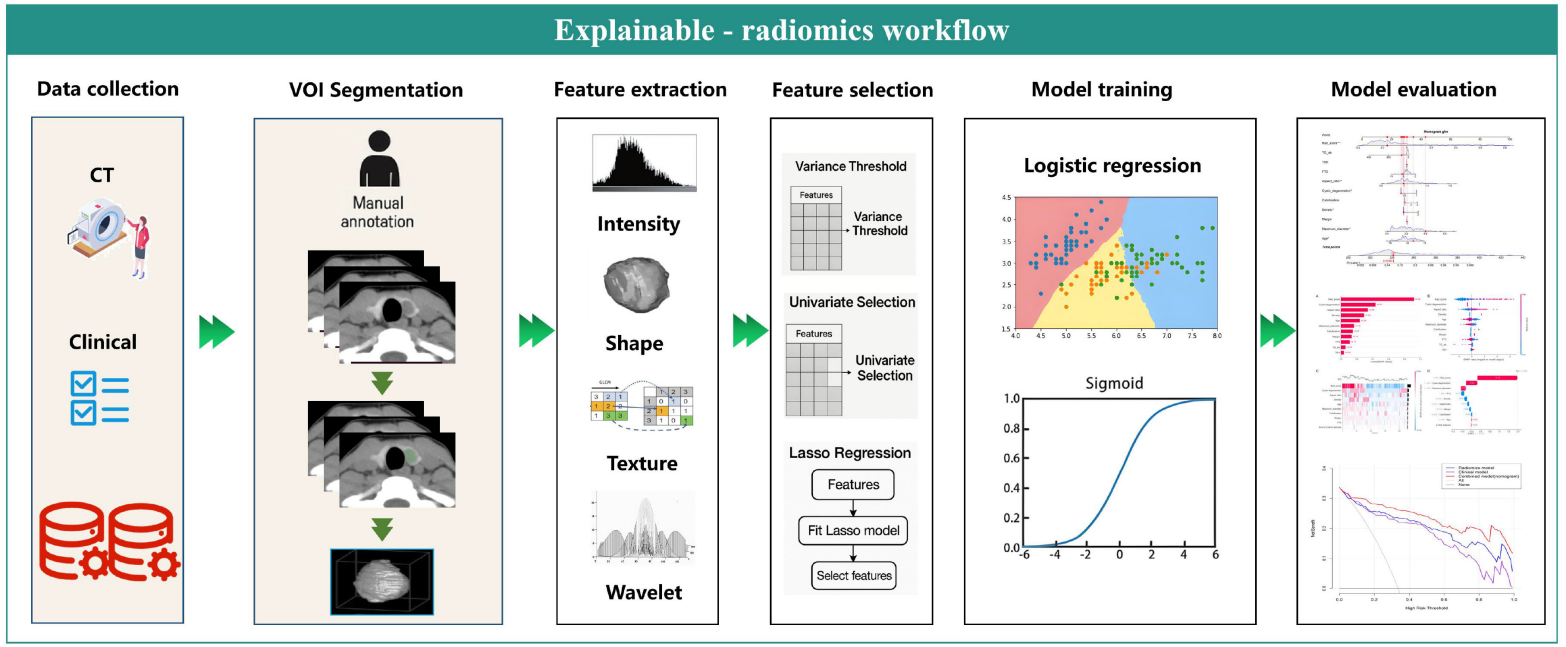
Interpretable radiomics workflow, divided into six parts: data collection, VOI segmentation, feature extraction, feature selection, model training, and model evaluation.

In this study, the key steps in constructing the radiomics workflow diagram are shown in **Figure 2**. CT images were imported into the Huiying (Radcloud Platform) platform to complete the automatic segmentation of lesions. A radiologist with 5 years of experience, unaware of the pathological results, manually delineated regions of interest along the margins of the primary thyroid lesion on the CT plain scan images layer by layer using the platform’s semi-automatic annotation tool. These regions were then confirmed by another radiologist with over 10 years of experience. In cases of disagreement, consensus was reached through discussion.

### 2.4 Image resampling and feature extraction

Before feature extraction, the Radcloud Platform automatically preprocesses the original images with the following parameters: resampling pixel spacing (1,1,1), binwidth = 15, interpolator sitkBSpline, and normalization = True. The normalization formula is:


$$f(x)=\frac{s(x-\mu_{x})}{\sigma_{x}}$$

where *x* is the original pixel intensity, *s*(*x*) is the normalized intensity, *μx* is the mean, and *σx* is the standard deviation.

Upon automatically extracting radiomic features from manually segmented tumor regions, one region of interest (ROI) was extracted for each patient, yielding a total of 1,409 features, which were divided into four categories: intensity features, describing pixel intensity information within the ROI (e.g., energy, entropy, mean, standard deviation, variance, maximum value, median, range, kurtosis, etc.); shape features, describing the shape and size of the ROI (e.g., volume, surface area, compactness, 2D/3D maximum diameter, flatness, etc.); texture features, describing the spatial relationships between pixels within the ROI (e.g., gray level co-occurrence matrix, gray level dependence matrix, gray level size zone matrix, gray level run length matrix, neighborhood gray tone difference matrix, etc.); and high-order features, which refer to features extracted from the transformed image after applying filtering transformations (such as logarithmic transformation, exponential transformation, wavelet transformation, etc.) to the image, including first-order features (i.e., intensity features) and texture features.

### 2.5 Feature selection

When constructing the model, centers 1 and 2 were designated as the model construction cohort, which was randomly divided into a training set (*n* = 229) and an internal validation set (*n* = 100) in a 7:3 ratio. Patients from center 3 were used as the external validation set, and the training set was used for radiomics feature selection. During feature selection, the variance threshold algorithm was first applied to select features, retaining those with a threshold greater than 0.8. The Select-K-Best algorithm was then used to further screen the features, retaining those with *P* < 0.05. Finally, 10-fold cross-validation LASSO regression analysis was employed to select the optimal features. The optimization objective of LASSO is:


$$y=(\frac{1}{2*n_{\text{samples}}})*y-Xw^{2}+alpha*w$$

In this equation, *X* is the feature matrix, *y* is the sample vector label, *n* is the number of samples, *w* is the vector regression model coefficient, and *α* is the LASSO penalty term.

Determine the optimal tuning parameter (λ) through MSE and obtain the non-zero coefficient features in the LASSO graph. Finally, select the non-zero coefficient feature linear mapping generated by the training queue LASSO model as Rad_score, and the calculation formula is:


$$\text{Rad\_score}=Intercept+\sum_{i=1}^{n}Coef_{i}\times Feature_{i}$$

Among them, Intercept is the intercept term of LASSO regression, *n* is the total number of features selected by LASSO, Coef_i_ is the LASSO coefficient of the i-th feature, and Feature_i_ is the i-th feature.

### 2.6 Model construction

Two algorithms, logistic regression (LR) and support vector machine (SVM), were used to construct three types of models—clinical models, radiomics models, and combined models (integrating clinical factors and Rad_score)—to systematically evaluate the discriminatory efficacy of different models for distinguishing between benign and malignant thyroid nodules. For the radiomics model, calculate the radiomics score (Rad_score) based on the selected optimal radiomics features and use it as an input variable to construct the model. For the clinical model, models were constructed using statistically significant clinical risk factors selected through univariate and multivariate logistic regression as input features. For the combined model, models were constructed using Rad_score and the aforementioned clinical risk factors as input variables. Among these, the combined model based on LR was further visualized as a column chart to enhance clinical practicality.

The LR algorithm was chosen for its good interpretability, allowing direct quantification of the contribution weights of each feature, making it suitable for constructing visualized column plots. The SVM algorithm was chosen for its advantages in handling high-dimensional features (such as radiomics features), enabling it to capture non-linear relationships between features through kernel functions and improve the classification performance of complex data. All models were parameterized on the training set to ensure comparability between different algorithms and models.

### 2.7 Model evaluation and SHAP analysis

The AUC of the ROC curve is used to evaluate the predictive performance of the model while providing the 95% confidence interval for the AUC. The model’s discriminative performance is quantified using metrics such as sensitivity, specificity, and accuracy. Calibration curves are used to validate the consistency between the model’s predicted probabilities and the actual occurrence probabilities. Decision curve analysis (DCA) is employed to quantify the net benefit of different models at different threshold probabilities, thereby assessing the clinical application value of the joint model. Validation is conducted using internal and external validation sets, with calibration curves used to indicate model reliability. Additionally, SHAP explainability techniques are employed to analyze the prediction process of the combined model, exploring the influence of different features on model decision-making.

### 2.8 Statistical analysis

Statistical analysis was performed using R software (Ver. 3.6.1, http://www.r-project.org). Continuous variables were first assessed for normality using the Kolmogorov–Smirnov test. Normally distributed data were analyzed using independent-samples *t*-tests and expressed as mean (standard deviation). Non-normally distributed data were analyzed using Mann–Whitney *U*-test and expressed as median [interquartile range]. Categorical variables were compared between groups using chi-square test and Fisher’s exact probability test. ROC curve analysis was used to calculate AUC, sensitivity, specificity, accuracy, and other metrics to evaluate the diagnostic performance of the model. A two-sided *P*-value <0.05 was considered statistically significant.

## 3 Results

### 3.1 Patient clinical information

A total of 370 patients who fully met the criteria were selected, with 229 cases in the training set (age 37–54 years, mean 47.39 ± 11.14 years, malignancy rate 33.7%), an internal validation set of 100 cases (age 38–55 years, mean 44.27 ± 10.83 years, malignancy rate 34.0%), and an external validation set of 41 cases (age 36–54 years, mean 46.74 ± 11.12 years, malignancy rate 53.7%). There were no significant differences among the three groups of patients in terms of relevant clinical risk factors (**Table 1**). The results of univariate and multivariate analyses showed significant differences in age, tumor maximum diameter, margin, density, cystic change, aspect ratio, and FT3 between benign and malignant nodules (*p* < 0.05) (**Table 2**).

**Table 1:** Demographic and clinical characteristics.

Characteristics	Training set (*n* = 229)	Internal validation set (*n* = 100)	External validation set (*n* = 41)	*P*-value
Benign/malignant				0.043
Benign	152 (66.4)	66 (66.0)	19 (46.3)	
Malignant	77 (33.6)	34 (34.0)	22 (53.7)	
Gender				0.955
Male	35 (15.3)	14 (14.0)	6 (14.6)	
Female	194 (84.7)	86 (86.0)	35 (85.4)	
Age (years)	48.00 (37.00-54.00)	47.00 (37.75-55.00)	43.00 (36.00–54.00)	0.081
Location				0.166
Right lobe	85 (37.1)	36 (36.0)	21 (51.2)	
Left lobe	70 (30.6)	34 (34.0)	16 (39.1)	
Both lobes	63 (27.5)	26 (26.0)	3 (7.3)	
Isthmus	11 (4.8)	4 (4.0)	1 (2.4)	
Number of nodules				0.078
1	150 (65.5)	65 (65.0)	35 (85.4)	
2	27 (11.8)	10 (10.0)	0 (0.00)	
>2	52 (22.7)	25 (25.0)	6 (14.6)	
Maximum diameter (cm)	2.40 [1.70–3.40]	2.20 [1.40–2.90]	2.10 [1.30–2.80]	0.014
Boundary				0.577
Clear	151 (65.9)	63 (63.0)	21 (51.2)	
Unclear	78 (34.1)	37 (37.0)	20 (48.8)	
Density				0.462
Homogeneous	178 (77.7)	78 (78.0)	14 (34.1)	
Heterogeneous	51 (22.3)	22 (22.0)	27 (65.9)	
Calcification				0.314
No calcification	180 (78.6)	84 (84.0)	35 (85.4)	
Microlithiasis	5 (2.2)	4 (4.0)	4 (9.8)	
Other Calcification	44 (19.2)	13 (13.0)	2 (4.9)	
Lobulation				0.661
Non-lobulated	187 (81.7)	89 (89.0)	10 (24.4)	
Lobulated	42 (18.3)	11 (11.0)	31 (75.6)	
Cystic Degeneration				0.838
Absent	86 (37.6)	46 (46.0)	17 (41.5)	
Present	143 (62.4)	54 (54.0)	24 (58.5)	
Aspect ratio	1.02 [0.93–1.14]	1.04 [0.95-1.20]	1.10 [0.96–1.21]	0.632
Platelets (×10^9^/L)	245.00 [202.00–295.00]	239.00 [191.75-278.50]	234.00 [205.00-285.00]	0.508
Neutrophils (%)	59.60 [52.90–65.90]	58.65 [55.27–64.15]	57.30 [53.20–62.10]	0.079
Lymphocytes (%)	31.10 [25.60–37.50]	32.15 [27.00–35.85]	30.80 [28.70–36.90]	0.085
Monocytes (%)	6.30 [5.30–7.40]	6.30 [5.60–7.30]	7.20 [6.20–7.80]	0.392
FT3 (pmol/L)	4.86 [4.36–5.38]	5.11 [4.61–5.41]	4.63 [3.93–4.94]	0.077
TT3 (nmol/L)	1.35 [1.08–1.78]	1.56 [1.17–1.98]	1.58 [1.39–1.84]	0.412
FT4 (pmol/L)	15.90 [14.12–17.88]	15.80 [14.20–17.45]	16.60 [15.30–18.30]	0.742
TT4 (nmol/L)	84.05 [7.61–118.25]	85.90 [7.75–107.50]	103.10 [93.70–114.70]	0.402
TSH (mIU/L)	1.91 [1.13–3.43]	2.47 [1.40–3.90]	103.10 [93.70–114.70]	0.086
TG-ab (%)	10.00 [3.75–15.36]	10.00 [3.03–16.95]	38.62 [14.15–65.39]	0.095

FT3, free triiodothyronine; TT3, total triiodothyronine; FT4, free thyroxine; TT4, total thyroxine; TSH, thyroid-stimulating hormone; TG-ab, thyroglobulin antibody.

**Table 2:** Univariate and multivariate analyses.

Characteristics	Univariate analysis				Multivariate analysis			
	OR	CI, lower	CI, upper	*P*-value	OR	CI, lower	CI, upper	*P*-value
Gender	0.966	0.517	1.861	0.915				
Age	0.951	0.933	0.970	**<0.001**	0.957	0.927	0.985	**0.004**
Location	0.720	0.552	0.932	0.064				
Number of nodules	0.729	0.544	0.963	0.089				
Maximum Diameter	0.129	0.079	0.198	**<0.001**	0.223	0.129	0.363	**<0.001**
Boundary	5.056	3.117	8.305	**<0.001**	2.433	1.201	4.957	**0.014**
Density	4.663	2.722	8.121	**<0.001**	3.180	1.422	7.262	**0.005**
Calcification	0.863	0.628	1.166	**0.049**	1.350	0.840	2.150	0.210
Lobulation	0.812	0.420	1.510	0.521				
Cystic Degeneration	0.107	0.063	0.178	**<0.001**	0.447	0.215	0.934	**0.031**
Aspect ratio	125.102	29.835	591.489	**<0.001**	9.067	1.516	59.027	**0.018**
Platelets	1.000	0.997	1.003	0.863				
Neutrophils	1.020	0.997	1.044	0.089				
Lymphocytes	0.980	0.954	1.006	0.131				
Monocytes	0.939	0.819	1.073	0.355				
FT3	0.769	0.585	0.99	**0.041**	0.620	0.420	0.940	**0.02**
TT3	0.743	0.476	1.106	0.166				
FT4	1.009	0.98	1.044	0.534				
TT4	0.998	0.994	1.002	0.296				
TSH	1.105	1.004	1.220	**0.043**	1.140	0.981	1.322	0.083
TG-ab	1.004	1.001	1.010	**0.047**	1.000	1.000	1.010	0.460

FT3, free triiodothyronine; TT3, total triiodothyronine; FT4, free thyroxine; TT4, total thyroxine; TSH, thyroid-stimulating hormone; TG-ab, thyroglobulin antibody. The boldfaced entries represent variables with statistical significance in univariate or multivariate analysis (P<0.05).

### 3.2 Results of feature selection

**Figure 3:**
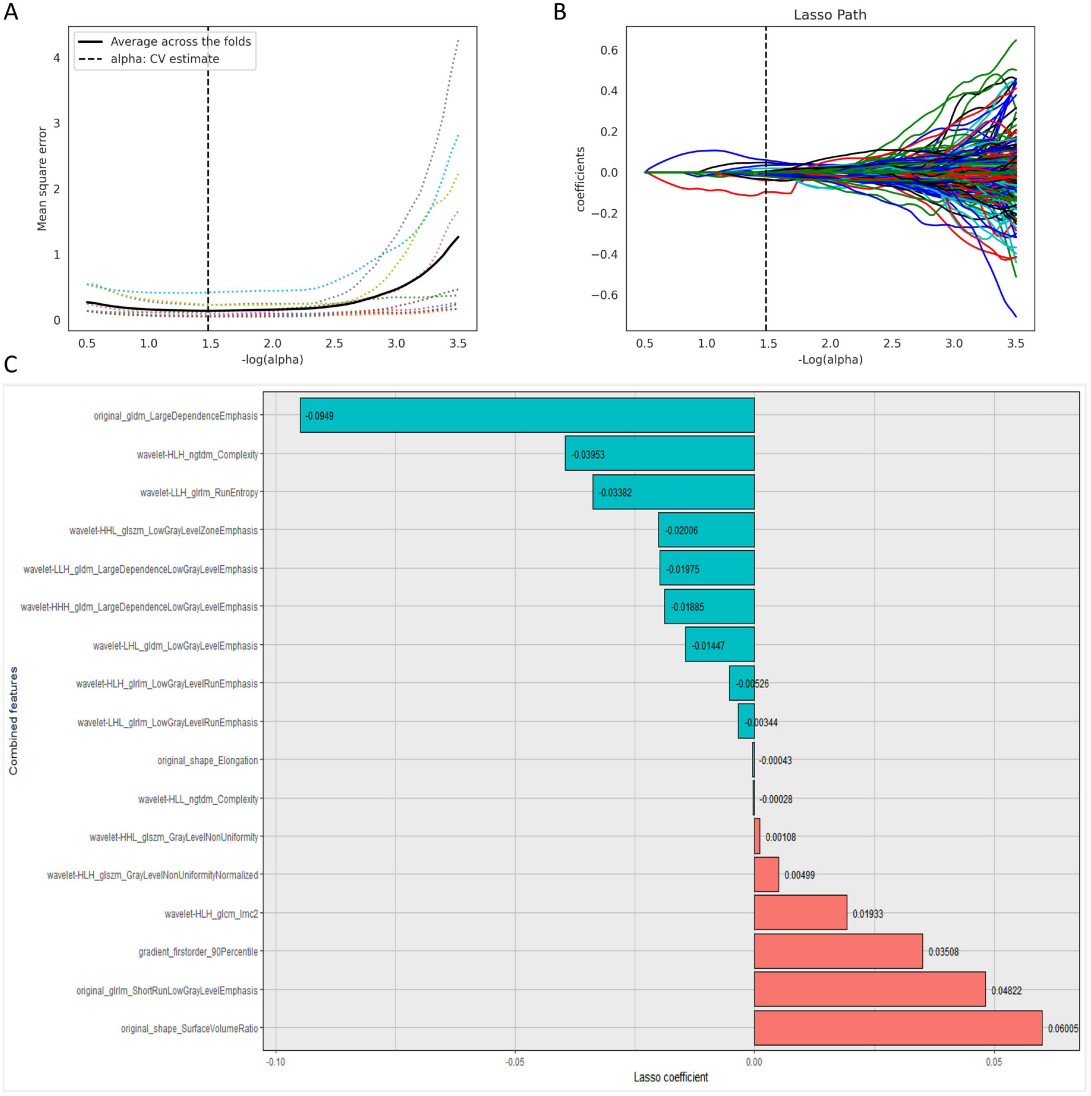
Radiomics feature selection diagram. **(A)** Mean square error coefficient determination diagram of the 10-fold cross-validation Lasso model. **(B)** Calculation diagram of the optimal radiomics feature coefficients (when -log(alpha) is 1.5, the MSE value is the lowest). **(C)** Final selected radiomics feature coefficient diagram. Firstorder, first-order feature; Shape: two shape features; GLDM, grayscale dependent matrix; NGTDM, neighborhood gray difference matrix; GLRLM, grayscale run-length matrix; GLCM, grayscale co-occurrence matrix; GLSZM, grayscale size zone matrix.

A total of 1,409 features were extracted from the VOI of each patient, and after screening, 17 features and their coefficients were determined (**Figure 3**). Coefficients can be positive or negative: a positive coefficient indicates a positive correlation between the feature and thyroid nodule malignancy, while a negative coefficient indicates a negative correlation. Among these, the coefficient for original_shape_SurfaceVolumeRatio (shape surface-area-to-volume ratio) is 0.06005, which is relatively high among positive coefficients, suggesting that it has a significant positive effect on assessing nodule malignancy. The coefficient for original_gldm_LargeDependenceEmphasis (grayscale dependence matrix large dependence emphasis) is -0.0949, with the largest absolute value among the negative coefficients, indicating that it has a strong indicative role in determining benign nodules. The absolute values of the coefficients for different features reflect their relative importance in the model, collectively forming the radiomics model for predicting the benign or malignant nature of nodules.

### 3.3 Model evaluation

The diagnostic performance of all models on the training set, internal validation set, and external validation set is shown in **Table 3**. Overall, the combined model (integrating clinical factors and Rad_Score) demonstrated superior predictive performance compared to standalone radiomics models and clinical models, with the combined model built using the logistic regression (LR) algorithm performing best across all datasets.

**Table 3:** Performance evaluation of all models.

Set	Model	Classifier	AUC (95% CI)	ACC	SEN	SPE
Training	Combined	LR	0.962 (0.946–0.983)	0.904	0.870	0.921
	Combined	SVM	0.953 (0.926–0.975)	0.895	0.857	0.914
	Rad_score	LR	0.934 (0.908–0.969)	0.873	0.792	0.914
	Rad_score	SVM	0.945 (0.913–0.974)	0.913	0.857	0.941
	Clinical	LR	0.921 (0.888–0.946)	0.847	0.831	0.855
	Clinical	SVM	0.943 (0.918–0.963)	0.869	0.753	0.928
IV	Combined	LR	0.913 (0.867–0.953)	0.850	0.853	0.848
	Combined	SVM	0.885 (0.811–0.942)	0.830	0.706	0.894
	Rad_score	LR	0.868 (0.811–0.922)	0.790	0.765	0.803
	Rad_score	SVM	0.843 (0.762–0.911)	0.810	0.794	0.818
	Clinical	LR	0.906 (0.837–0.958)	0.850	0.794	0.879
	Clinical	SVM	0.848 (0.773–0.907)	0.780	0.676	0.833
EV	Combined	LR	0.914 (0.83–0.975)	0.805	0.909	0.684
	Combined	SVM	0.842 (0.713–0.941)	0.780	0.818	0.737
	Rad_score	LR	0.821 (0.682–0.931)	0.707	0.682	0.737
	Rad_score	SVM	0.811 (0.652–0.933)	0.829	0.864	0.632
	Clinical	LR	0.88 (0.775–0.951)	0.732	0.864	0.579
	Clinical	SVM	0.868 (0.765–0.952)	0.756	0.773	0.737

T, training set; IV, internal validation set; EV, external validation set; AUC, area under the curve; ACC, accuracy; CI, confidence interval; SEN, sensitivity; SPE, specificity.

**Figure 4:**
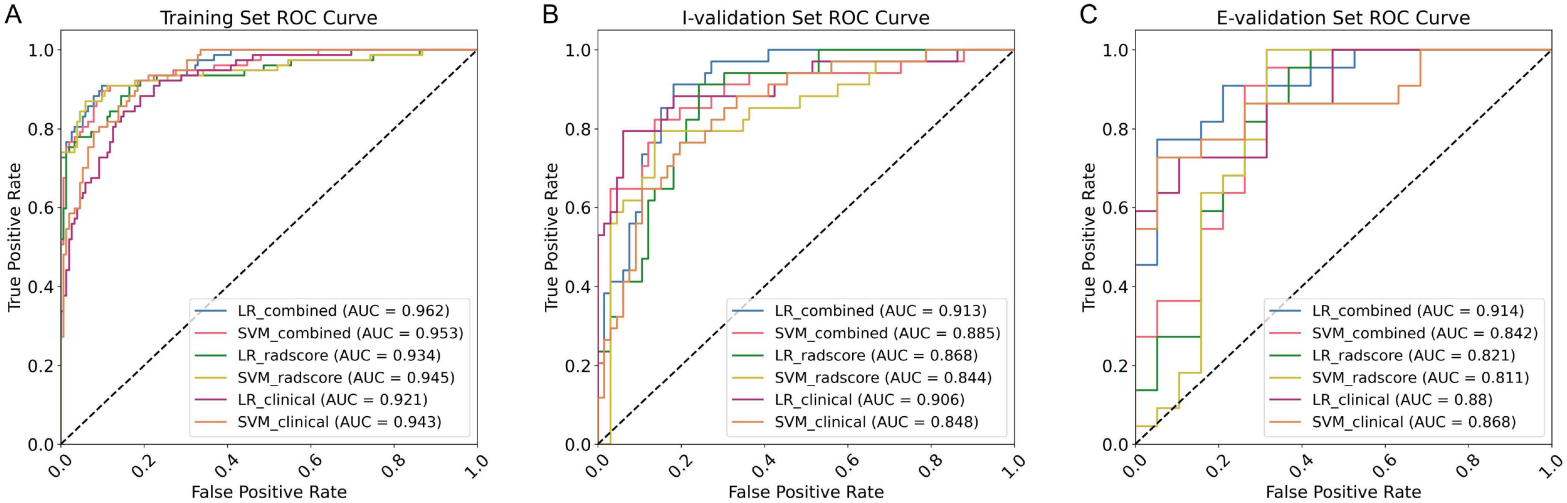
Prediction performance of the radiomics model in the training set **(A)** and internal **(B)** and external **(C)** validation sets. I-Validation, internal validation set; E-Validation, external validation set.

In the training set, the AUC of the LR combined model was 0.962 (95% CI: 0.946–0.983), significantly higher than that of the radiomics model (AUC = 0.934) and clinical model (AUC = 0.921) using the same algorithm and also superior to the SVM combined model (AUC = 0.953); its accuracy (ACC = 0.904), sensitivity (SEN = 0.870), and specificity (SPE = 0.921) all remained at high levels. Although the radiomics model using the SVM algorithm achieved a high AUC (0.945) in the training set, its performance declined more significantly in the validation set. The results of the internal validation set showed that the LR combined model still maintained the best performance (AUC = 0.913, 95% CI: 0.867–0.953), an improvement of approximately 3.2% compared to the SVM combined model (AUC = 0.885), while the AUC of the standalone imaging-based model and clinical model were both below 0.90 (LR imaging-based model = 0.868 and LR clinical model = 0.906). In the external validation set, the AUC of the LR combined model was 0.914 (95% CI: 0.830–0.975), significantly higher than that of the SVM combined model (AUC = 0.842) and all standalone models (AUC range: 0.811–0.880), demonstrating stronger generalization ability. Notably, although the SVM radiomics model had a high accuracy (0.829) in the external validation set, its specificity was only 0.632, suggesting a risk of overfitting; in contrast, the LR combined model maintained a more balanced performance between sensitivity (0.909) and specificity (0.684). The ROC curve (**Figure 4**) visually demonstrates that the LR combined model’s curve consistently outperforms other models across all datasets, further confirming its superiority in distinguishing benign from malignant thyroid nodules.

### 3.4 Nomogram construction

**Figure 5:**
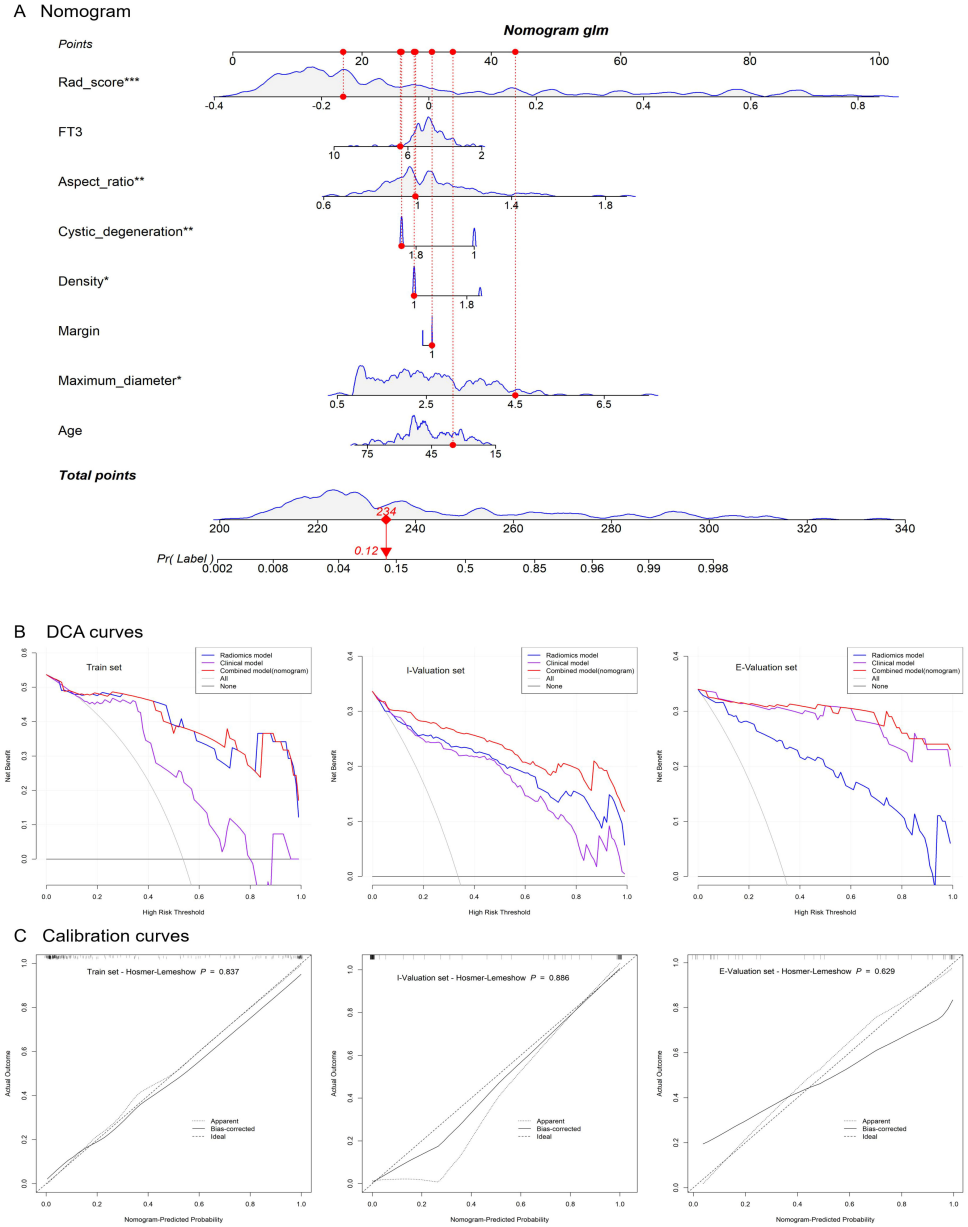
Nomogram **(A)**, DCA curves **(B)**, and calibration curves **(C)**.

To further enhance the clinical utility of the model, a column chart was constructed using a joint model based on radiomics scores (Rad_score), FT3, aspect ratio, cystic degeneration, density, margin, maximum diameter, and age (**Figure 5A**). By mapping each feature to the corresponding scale axis and the “Total points” axis, the malignant risk probability of thyroid nodules for individual patients can be intuitively calculated, providing a quantitative reference for clinical decision-making. The decision curve analysis (DCA, **Figure 5B**) shows that in the training set, internal validation set, and external validation set, the net benefit of the LR joint model was significantly superior to the “All” (assuming all samples are malignant) and “None” (assuming all samples are benign) strategies and maintained a stable advantage in the high-risk threshold range of 0.1–0.9, indicating that the model has high value in clinical applications (e.g., assisting in determining whether a biopsy is necessary). The calibration curve results (**Figure 5C**) show that the LR combined model exhibits a good fit between predicted and actual probabilities in the training set (Hosmer–Lemeshow test, *P* = 0.937), internal validation set (*P* = 0.806), and external validation set (*P* = 0.629), with curves approaching the ideal diagonal line. This result confirms from a calibration perspective that the LR combined model not only exhibits excellent discriminative ability (AUC performance) but also provides probability predictions that are more reliable and better suited to clinical needs.

### 3.5 SHAP analysis

**Figure 6:**
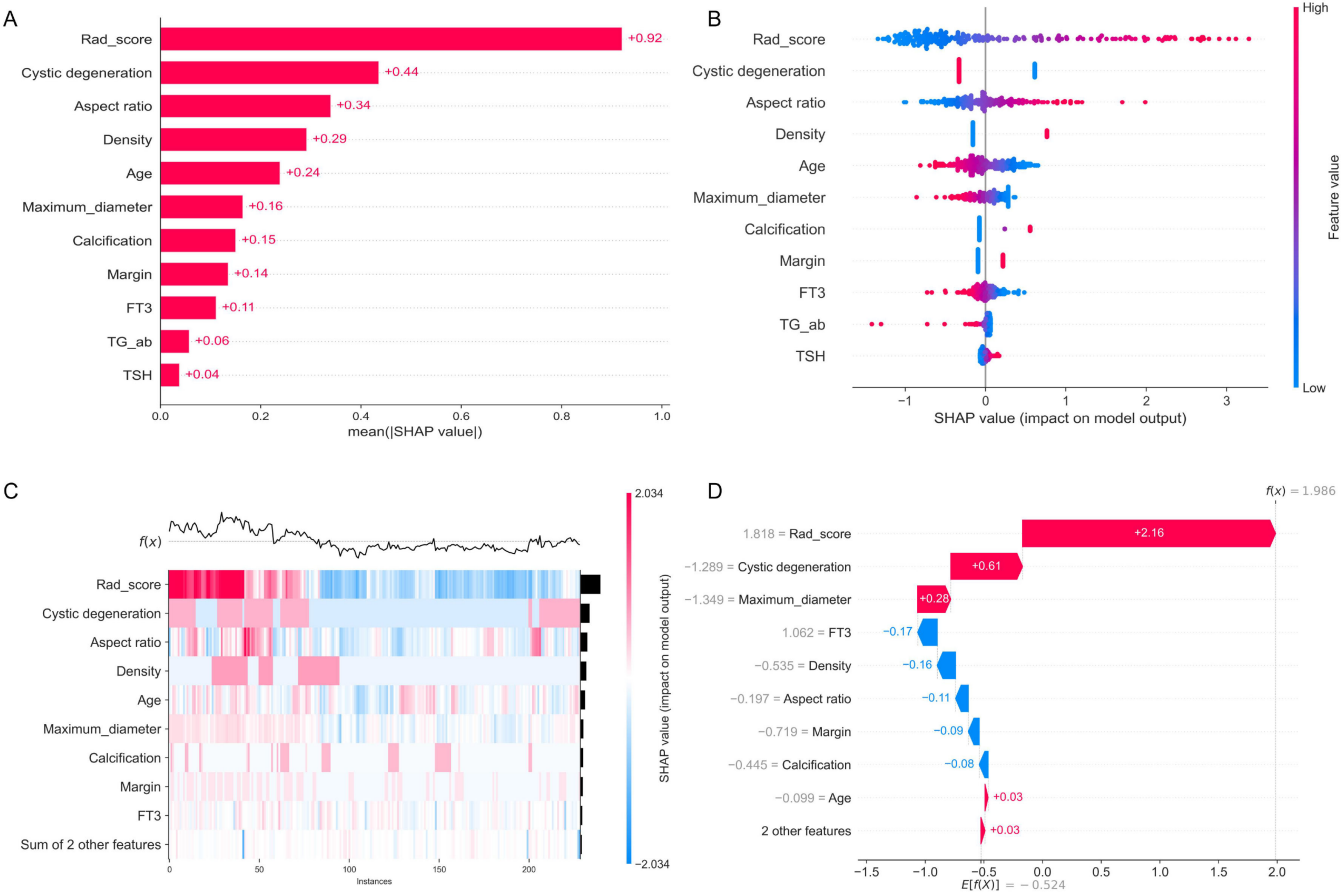
SHAP analysis results. Feature weight bar plot **(A)**, feature value beeswarm plot **(B)**, heatmap plot **(C)**, and waterfall plot **(D)**.

We conducted an interpretability study of the model using SHAP interpretability analysis, aiming to quantify and visualize the contribution of each feature in the joint model to the prediction of benign or malignant thyroid nodules. The SHAP histogram (**Figure 6A**) shows that the Rad_Score has the highest average absolute SHAP value (0.92), making it the feature with the greatest influence on model predictions, while TSH (0.04) and TG-ab (0.06) have relatively weaker influences. The SHAP swarm plot (**Figure 6B**) further illustrates the association pattern between feature values and SHAP values: high Rad_Score values correspond to positive SHAP values, indicating that an increase in Rad_Score significantly increases the predicted probability of malignant nodules; high cystic change values are associated with negative SHAP values, suggesting that this feature tends to indicate benign nodules. Additionally, increased aspect ratio and unclear borders are both associated with positive SHAP values, indicating that these features are positively correlated with malignant risk.

The SHAP heatmap sorted by sample prediction values (*f*(*x*)) (**Figure 6C**) presents the global distribution characteristics of feature values and their SHAP influences: in samples with higher prediction values (tending toward malignancy), high Rad_Score values are highly consistent with strong positive SHAP influences; in samples with lower prediction values (tending toward benignity), the negative influences of cystic changes and maximum diameter are significant. The SHAP waterfall plot (**Figure 6D**) illustrates the specific influence path of features on the prediction results using a single sample as an example. The baseline predicted value (*E*[*f*(*X*)]) for this sample is -0.524, and the final predicted value is 1.986, primarily driven by the positive contribution of Rad_Score (+2.16). Meanwhile, cystic changes (-1.289) and maximum diameter (-1.349) exert negative influences on the probability of malignancy, intuitively reflecting the synergistic effects among features.

## 4 Discussion

Literature indicates that approximately 65% of the general population can be detected with thyroid nodules, with only 5%–15% being malignant lesions. Accurate differentiation between benign and malignant nodules is critical for making individualized clinical decisions (7, 17). Notably, unlike most previous relevant studies that primarily utilized contrast-enhanced CT, the present study developed a clinical imaging informatics combined model based on plain CT images to address the challenge of distinguishing benign from malignant thyroid nodules with a diameter ≥1 cm. Furthermore, prior related studies lacked model explainable analysis, whereas we performed SHAP explainable analysis on the optimally performing model and conducted detailed analysis and discussion on the predictive contributions of Rad_score and clinical risk factors to the model as well as the prediction process—this significantly enhanced the interpretability of our model. Ultimately, the LR combined model established in this study achieved an AUC of 0.914 for distinguishing benign from malignant lesions in the external validation set, which was significantly superior to traditional imaging examinations.

Ultrasound, owing to its ease of operation and non-invasive, radiation-free nature, has become the preferred screening method for thyroid nodules (18). Existing ultrasound radiomics studies have demonstrated significant efficacy in distinguishing challenging TI-RADS grades 3–5 nodules, with reported AUC values ranging from 0.841 to 0.975 (19–23). However, this technique is highly operator-dependent, with variations in image acquisition and analysis potentially introducing significant bias that limits model generalizability. Compared to ultrasound, PET/CT and MRI possess inherent limitations in their clinical applicability. PET/CT not only carries higher costs but also exhibits relatively lower diagnostic efficacy (reported AUC values of 0.736, 0.757, and 0.818) (24–26), all significantly below the AUC value of 0.914 achieved by the model in this study. Although MRI offers superior soft tissue contrast and models constructed from MRI demonstrate better performance than PET/CT (27, 28), its clinical application remains constrained.

Enhanced CT radiomics has garnered attention for providing richer tumor hemodynamic information, with reported AUC values ranging from 0.923 to 0.960 (29–31). Notably, the combined model developed in this study based on plain CT scans achieved an AUC of 0.914 while demonstrating a diagnostic performance comparable to that of contrast-enhanced CT. This highlights several unique advantages of plain CT: in terms of image quality, it effectively avoids the blurring of lesion boundaries caused by overlapping tissue enhancement patterns seen in contrast-enhanced scans, thereby significantly improving segmentation consistency and reliability. In terms of feature stability, it eliminates variability introduced by inconsistent contrast agent injection parameters, enhancing the reproducibility and generalizability of the radiomics model; regarding clinical applicability, this approach maintains excellent diagnostic performance while substantially reducing radiation exposure and simplifying examination procedures, rendering it more suitable for large-scale screening and routine follow-up in practical clinical settings.

In this study, 17 key features were ultimately selected from the radiomics features for model construction. For features with positive coefficients, original_shape_SurfaceVolumeRatio (the ratio of surface area to volume of the shape) has a relatively large weight coefficient. A relatively large surface-area-to-volume ratio indicates that the lesion has a more irregular shape, which may be related to the fact that malignant thyroid nodules typically grow in a more disordered manner in clinical practice. Therefore, a higher value of this feature is more likely to indicate a malignant nodule, which aligns with our research objective of using imaging features to determine the nature of nodules. original_glrlm_ShortRunLowGrayLevelEmphasis (short run low gray level emphasis in the gray run length matrix) also has a relatively high positive coefficient, which may indicate that short runs with low gray values are more prominent in malignant nodules, reflecting increased disorder and heterogeneity in the internal tissue structure of the nodule, thereby aiding in the identification of malignant nodules. Among features with negative coefficients, original_gldm_LargeDependenceEmphasis (large dependence emphasis in the gray dependence matrix) has the largest absolute value; benign nodules may have more regular tissue structures, leading to more stable gray dependence relationships and large dependence characteristics. Therefore, higher values of this feature are more likely to indicate benign nodules, which is of significant importance for determining the benign nature of nodules. The wavelet-HLH_ngtdm_Complexity (complexity of the wavelet-transformed HLH directional neighborhood gray-level difference matrix) has a negative coefficient, which may indicate that the complexity of neighborhood gray-level differences is relatively low in benign nodules, reflecting the relative uniformity of the nodule’s internal tissue structure; this aligns with our research objective of distinguishing between benign and malignant nodules. These key features reflect the relationship between the imaging characteristics of thyroid nodules and their benign or malignant nature from different perspectives. By analyzing and integrating these features, the accuracy of the model in predicting the benign or malignant nature of thyroid nodules can be improved.

SHAP interpretability analysis provides an intuitive and quantitative validation of the predictive logic of the joint model, significantly enhancing the clinical credibility of the model. As shown in the SHAP bar chart, Rad_score, as a comprehensive quantitative indicator of radiomics features, contributes most to the prediction of malignant nodules, confirming the core value of radiomics features in capturing tumor heterogeneity, which is consistent with previous studies indicating that radiomics can effectively extract tumor microfeatures invisible to the naked eye. The feature correlation patterns revealed by the SHAP honeycomb plot further reinforce the consistency between the model’s predictions and clinical pathological logic. The positive influence of Rad_score is directly related to common radiological phenotypes in malignant nodules (such as texture disorder and irregular shape), while the negative influence of cystic changes aligns with the pathological features of cystic degeneration in benign nodules, supporting the clinical understanding that “cystic changes are an important marker of benign thyroid nodules.” SHAP waterfall plots successfully reveal a glimpse into the model’s “black box” mechanism by analyzing the decision-making path for individual samples—for example, in a case ultimately diagnosed as malignant, despite its small maximum diameter (typically considered a low-risk indicator), the model correctly predicted malignancy because Rad_score (reflecting internal structural disorder) was significantly elevated. This aligns closely with clinical experience that “small, irregularly shaped nodules may still be malignant,” highlighting the model’s adaptability to complex clinical scenarios. The SHAP analysis reveals the model’s decision-making mechanisms across multiple dimensions. The waterfall plot (**Figure 6D**) deconstructs the prediction pathway at the individual case level, while the heatmap (**Figure 6C**) validates the stability of feature influences from a global perspective: in samples with high malignant probability, features such as Rad_score and aspect ratio consistently exert positive driving forces; conversely, in benign samples, markers like cystic changes exert a significant negative influence. This high consistency between radiomics features and clinical–pathological logic enhances the credibility of the model’s decisions. More importantly, the interpretability framework established in this study transcends mere performance improvement, becoming pivotal for advancing AI’s clinical implementation. Firstly, it transforms “black-box” decision-making into a transparent, traceable chain of logic. When clinicians can intuitively observe that Rad_score is the core driver and understand why certain benign features (such as cystic changes) are overruled in specific cases, it fosters substantive trust in the model. Secondly, it provides a quantifiable communication tool for clinical decision-making. During multidisciplinary consultations or doctor–patient discussions, clinicians can use visualized results to intuitively explain the rationale behind decisions regarding biopsy or follow-up, enhancing the objectivity and persuasiveness of these decisions. Ultimately, it empowers clinicians to proactively identify model limitations. When feature contributions conflict or diverge significantly from clinical judgement, the system’s transparency prompts physicians to conduct more cautious, comprehensive evaluations, avoiding blind adherence. Thus, this model not only delivers predictions but constructs a human–machine collaborative decision-making system capable of engaging in dialogue with clinical knowledge and mutual validation. This lays a robust foundation for its eventual transformation into a reliable clinical tool.

### 4.1 Limitations of this study and corresponding solutions

Firstly, the retrospective design may introduce selection bias, necessitating prospective multicenter studies in the future. Secondly, the sample size (particularly in the external validation cohort) remains insufficient and requires expansion. Thirdly, due to segmentation challenges with small or ill-defined nodules, only lesions ≥1.0 cm in diameter were included, potentially limiting the generalizability of findings. Finally, variations in multi-center CT acquisition and reconstruction parameters (e.g., slice thickness, tube current, kernel function) exerted dual effects: while objectively validating the model’s strong generalization capability, they inherently constrained radiomics feature reproducibility, creating a bottleneck for standardized application. Future research must prioritize establishing prospective standardized CT scanning protocols. This concerns not merely parameter uniformity but is fundamental to controlling data quality at source and addressing the challenge of radiomics “feature stability”. Adherence to guidelines such as the Radiomics Quality Score (RQS) and the Imaging Biomarker Standardisation Initiative (IBSI) constitutes the core prerequisite for ensuring reliable model replication and eventual deployment across different healthcare institutions.

In the future, we can further explore the integration of imaging features from different modalities (such as ultrasound, MRI, PET-CT, etc.) with CT imaging features to construct a multi-modal imaging model, thereby improving diagnostic accuracy and specificity. Furthermore, biological information such as genetic testing and proteomics can be combined to deeply explore potential biomarkers for distinguishing benign from malignant thyroid nodules, providing a more robust biological foundation for radiomics models. Additionally, conducting large-scale prospective multicenter studies to validate the model’s effectiveness in different populations and clinical settings is an important direction for future research.

## 5 Conclusion

This study constructed a CT–clinical combined model based on multicenter data, integrating radiomics features with clinical risk factors, and demonstrated good performance in distinguishing the benignity or malignancy of thyroid nodules. By combining SHAP explainability technology to analyze the model’s decision-making process, it provides an accurate method for non-invasive diagnosis and holds promise for optimizing individualized clinical management.

## Data Availability

The inability to provide raw data primarily stems from ethical, privacy, and regulatory constraints inherent to medical research involving human subjects: Patient Privacy Protection: Raw data (including CT images with DICOM metadata, clinical records) contains direct or indirect identifiers (e.g., hospital IDs, precise imaging timestamps, combined demographic details) that could potentially identify individuals, violating privacy laws (e.g., the Declaration of Helsinki, Personal Information Protection Law) and breaching the trust of participants. Ethics Committee Restrictions: The study was approved by institutional review boards (IRBs) of the three participating centers under the condition that raw data would be strictly protected. IRB guidelines explicitly prohibit the sharing of unprocessed data to prevent re-identification risks, mandating only de-identified datasets (with identifiers removed) for academic use. Multi-center Data Agreements: The raw data is governed by data usage protocols signed across the three institutions, which restrict distribution of unaltered data to safeguard institutional and participant interests, limiting access to pre-approved, anonymized formats only. These constraints ensure compliance with ethical standards and legal requirements, prioritizing participant confidentiality over unrestricted data access. Requests to access the datasets should be directed to Yang Jing, Huiying Medical Technology Co., Ltd, Beijing, 100080, China. Phone: +87 18780057870. E-mail: 605413559@qq.com.
